# Predictors of Poor Outcomes in Critically Ill Adults with Hematologic Malignancy

**DOI:** 10.1155/2016/9431385

**Published:** 2016-02-24

**Authors:** Marion Cornish, Michael B. Butler, Robert S. Green

**Affiliations:** ^1^Department of Critical Care, Dalhousie University, Halifax, NS, Canada B3H 2Y9; ^2^Trauma Nova Scotia, Halifax, NS, Canada B3H 2Y9

## Abstract

*Background*. Patients with hematologic malignancy (HM) often require intensive care unit (ICU) admission due to organ failure through disease progression or treatment-related complications.* Objective*. To determine mortality and prognostic variables in adult patients with HM who were admitted to ICU.* Methods*. Structured chart review of all adult patients (age ≥ 18 years) with HM admitted to ICU of a Canadian tertiary care hospital between 2004 and 2014. Outcome measures included mortality (ICU, 30-day, 60-day, and 12-month). Logistic regression was performed to determine predictors of mortality.* Results*. Overall, there were 206 cases of HM admitted to the ICU during the study (mean age: 51.3 ± 13.6 years; 60% male). Median stay was 3 days, with 14.1% requiring prolonged ICU admission. ICU mortality was 45.6% and increased to 59.2% at 30 days, 62.6% at 60 days, and 74.3% at 12 months. Predictors of increased ICU mortality included mechanical ventilation requirement and vasopressor therapy requirement, while admission to ICU postoperatively and having myeloma were associated with decreased mortality.* Conclusions*. Patients admitted to ICU with HM have high mortality (45.6%), which increased to 74.3% at 1 year. Analysis of multiple variables identified critical illness, postsurgical admission, and myeloma as predictors of patient outcomes.

## 1. Introduction

Critical care medicine provides resuscitation and supportive care to a varied population, including patients with guarded prognosis. Patients with hematologic malignancy (HM) that require admission to the intensive care unit (ICU) for resuscitation and organ support often have poor outcomes, particularly those patients who have developed febrile neutropenia or undergone allogeneic stem cell transplant [1]. The most common reasons for ICU admission in this population include sepsis and respiratory failure [2, 3]. However, recent advances in hematologic care and the use of intensified treatment protocols have contributed to improving overall survival rates for critically ill HM patients [4]. Despite this, controversy exists over the potential benefit versus the medical futility of providing critical care to this patient population and whether the use of ICU resources for patients with a HM is appropriate [5].

Mortality in patients with HM admitted to the ICU varies from 33% to 69% in some studies [6, 7], with reported 5-year survival rates of 17–20% [2, 8]. Factors associated with ICU mortality include multiorgan failure [9], mechanical ventilation [10], and the use of vasopressors [11]. Unfortunately, prognostic factors vary depending on the characteristics of the study center, patient population, type of HM, and, in some cases, conditioning regime. Although a number of studies have investigated long-term outcomes in HM patients admitted to the ICU [12–15], additional studies from different centers are needed to confirm the findings of earlier reports. Specifically, there is little data available from the Canadian health care system to guide the care of critically ill patients with HM [13, 16].

The objective of this study was to describe mortality and investigate factors predictive of poor outcomes in critically ill adults with HM who required ICU admission at a Canadian tertiary care center.

## 2. Methods

### 2.1. Setting

This study was performed at the Nova Scotia Health Authority, Victoria General Hospital (VGH) site in Halifax, Nova Scotia, Canada. The VGH ICU is an 11-bed medical-surgical ICU which is staffed by board certified intensivists and is the primary ICU supporting patients with hematologic malignancies in the province of Nova Scotia. Approval for this study was obtained from the Nova Scotia Health Authority Research Ethics Board.

### 2.2. Study Design

A retrospective unblinded review of patient medical records was conducted. We reviewed the medical records of all adult (age ≥ 18 years) patients with known diagnosis of a HM who were admitted to the VGH ICU between January 1st 2004 and December 31st 2014.

### 2.3. Participants

All patients with a known diagnosis of acute leukemia, chronic leukemia, lymphoma, or multiple myeloma were considered for inclusion. Patients with a HM that did not fit any of these diagnostic criteria were considered for inclusion and placed in the category of “other.” Study participants were identified through a manual review of a dedicated paper-based ICU admission log. Chart reviews were performed by reviewing the medical record of each study participant and abstracting information into a database developed by the investigative team. If a patient had more than one ICU admission during the same hospital admission, data from their first admission was included in the analysis to ensure the independence of observations. The study team reached consensus that a period of two years between ICU admissions would be a sufficient amount of time to demarcate different observations for the same patient. Thus, data for patients admitted to the ICU on separate occasions more than two years apart were considered to be separate data sets.

### 2.4. Data Collection

#### 2.4.1. Variables

We collected patient demographics, ICU admission diagnosis, length of ICU stay, occurrence of ICU readmission, type of HM, bone marrow transplant and type (autologous versus allogeneic, matched or unmatched), presence of graft versus host disease, venoocclusive disease, or febrile neutropenia, sepsis or steroid use, and the indication for use of steroids. The presence of anemia, neutropenia, or thrombocytopenia on admission to the ICU and the presence of infection either prior to or during ICU admission were collected as laboratory variables. The presence of infection was included if there was documentation of positive cultures in blood, urine, sputum or bronchial washings, wound swabs, or catheter tips. The requirement for organ support was documented by the use of vasopressors and inotropes, mechanical ventilation, and dialysis during the ICU stay. Only data available in the medical record was abstracted. Data that was not present was coded as not available; no data was imputed.

#### 2.4.2. Definitions

The categorization of patients was performed by the investigators based on what was deemed the most significant issue requiring ICU admission. The following terms were used to categorize ICU admission diagnosis: respiratory, neurological, hemodynamic, renal, postoperative, and follow-up postcardiopulmonary arrest. “Respiratory” was defined as the requirement of respiratory support including noninvasive mechanical ventilation and/or invasive mechanical ventilation. “Neurological” included patients who had a decreased level of consciousness as documented in the patient chart on admission to ICU for various reasons (e.g., seizure and cerebrovascular incident). “Renal” was defined as ICU admission for the requirement of renal replacement therapy during ICU admission (chronic renal replacement therapy excluded). “Hemodynamic” was defined as the need for ICU admission for hypotension necessitating vasopressor and/or inotropic support, or for invasive monitoring that could not be provided while on a ward setting. “Postoperative” was defined as patients requiring ICU admission following an operative procedure necessitated by a complication of their HM. “Follow-up postcardiopulmonary arrest” included patients admitted to the ICU following an arrest on the ward.

### 2.5. Outcomes

The primary outcome of this study was ICU mortality. Secondary outcomes included 30-day, 60-day, and 12-month mortality.

### 2.6. Data Analysis

Baseline characteristics of included patients were assessed using descriptive statistics (proportions, means, and standard deviations) in terms of age, sex, type of HM, diagnosis, ICU length of stay, ICU readmission, prior stem cell transplant, steroid use prior to ICU admission, and presence of anemia on ICU admission, graft versus host disease, neutropenia, febrile neutropenia, thrombocytopenia, or infection. A logistic regression model was used to assess the association between patient factors and outcome (mortality). We compared outcomes in patients with acute leukemia, chronic leukemia, lymphoma, or myeloma using patients with any other form of HM as the reference. Testing the effect of the logistic regression model was performed using Wald's Test. All tests were two-sided and a *P* value < 0.05 was considered to be statistically significant. To assess the goodness-of-fit of the models, we report the Hosmer-Le Cessie test result using a *P* value of greater than 0.05 as a satisfactory fit.

## 3. Results

### 3.1. Patient Characteristics

**Figure 1:**
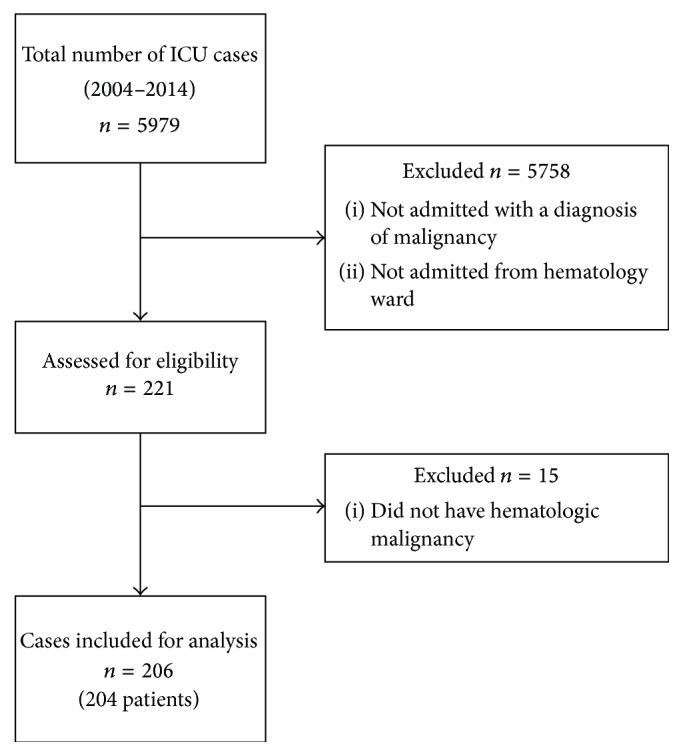
Selection of study participants.

A flow chart outlining the selection of study participants is shown in Figure 1. There were 204 adult patients with HM admitted to the ICU between 2004 and 2014. Two of these patients were admitted on two different occasions greater than two years apart, with each of these ICU admissions considered a separate case. Thus, overall there were 206 cases of HM requiring ICU admission during the study period. The characteristics of these 206 cases are shown in Table 1. The mean patient age was 51.3 ± 13.6 years and 59.7% (123/206) were male. The median duration of ICU stay was 3 days (interquartile range: 6 days). The most common types of HM seen were acute leukemia (71/206; 34.5%) and lymphoma (69/206; 33.5%). The most common ICU admission diagnoses were respiratory failure (82/206; 39.8%) and hemodynamic instability (79/206; 38.3%). In less than half of cases of HM patients admitted to the ICU, the patient had received a previous stem cell transplant (92/206; 44.7%).

### 3.2. Intensive Care Interventions

Interventions during the course of HM patients in the ICU are shown in Table 2. Intubation and mechanical ventilation were required in 148 cases (71.8%). Vasopressor therapy was required in 117 cases (56.8%) and hemodialysis was necessary in 36 cases (17.4%).

### 3.3. Patient Outcomes

Table 3 reports outcomes for HM patients admitted to the ICU. Mortality during ICU admission was 45.6% (94/206), compared to an average mortality of 11.8% for all patients admitted to this ICU during the study period. Of cases where HM patients survived their ICU stay, 59.2% (122/206) died at 30 days and one-year mortality was 74.3% (153/206).

Mortality outcomes for patients with different types of HM are shown in Table 4. Patients admitted to the ICU with chronic leukemia had the highest rate of ICU mortality at 64.3% (9/14). The lowest rate of ICU mortality was seen in patients admitted with multiple myeloma (4/21; 19.1%). Patients who had undergone a previous stem cell transplant had an ICU mortality of 43.5% (40/92) and a 12-month mortality of 66.3% (61/92).

### 3.4. Prognostic Factors

A logistic regression model was used to identify predictors of poor outcomes for HM patients admitted to the ICU (Table 5). Based on logistic regression analysis, the use of vasopressors (adjusted estimate [AE] 3.73 [95% CI 1.71 to 8.14]; *P* < 0.001) and mechanical ventilation (AE 3.42 [95% CI 1.39 to 8.44]; *P* = 0.008) in the ICU were associated with increased ICU mortality. The use of vasopressors continued to be associated with 30-day mortality (AE 2.21 [95% CI 1.05 to 4.63]; *P* = 0.036) and 60-day mortality (AE 2.14 [95% CI 1.01 to 4.55]; *P* = 0.047), but not mortality at 12 months. The Hosmer-Le Cessie test for all of the logistic regression models was nonsignificant, indicating no significant problems with the fit of the models. Additionally, all variance inflation factors were less than two, showing no significant difficulty with colinearity in the predictors.

Having a previous stem cell transplant was associated with decreased mortality at 12 months (AE 0.46 [95% CI 0.21 to 0.99]; *P* = 0.047) compared with patients who did not have a prior stem cell transplant. We evaluated mortality outcomes in patients with acute leukemia, chronic leukemia, lymphoma, or myeloma, using patients with any other type of HM as a reference. The only type of HM that was associated with improved outcomes was multiple myeloma. A diagnosis of myeloma on admission was associated with decreased mortality at 30 days (AE 0.25 [95% CI 0.07 to 0.95]; *P* = 0.041), 60 days (AE 0.22 [95% CI 0.06 to 0.85]; *P* = 0.028), and 12 months (AE 0.18 [95% CI 0.05 to 0.72]; *P* = 0.015). Being admitted to ICU following an operation that was necessitated by a complication of HM was associated with decreased mortality in the ICU (AE 0.21 [95% CI 0.05 to 0.86]; *P* = 0.031) and at 12 months (AE 0.15 [95% CI 0.04 to 0.61]; *P* = 0.008).

## 4. Discussion

This study represents one of the largest Canadian-based evaluations of outcomes in HM patients who are admitted to the ICU. The results of this 10-year retrospective review demonstrate a high mortality rate (45.6%) in cases of HM patients who required ICU admission compared with 11.8% in the general population in this ICU. Interestingly, in cases of HM patients who survived ICU admission, mortality rates were found to increase to 74.3% after 1 year. Despite our evaluation of multiple variables in an effort to estimate patient prognosis on ICU admission, only the requirement of vasopressor and mechanical ventilation were associated with increased ICU mortality, whereas a postoperative admission and multiple myeloma were associated with increased survival. Factors such as patient age, gender, and admission diagnosis were not predictive of mortality in our study population.

Based on the results of the available data, ICU and in-hospital mortality rates remain high in this patient population [12, 17–22]. Reported mortality has ranged from 33.7% to 84.1% for patients with HM who require ICU admission [6, 19]. Because of this relatively high rate of mortality, some authors have suggested that admission of these patients to the ICU may not be beneficial [19, 23]. A study in 1993 demonstrated that there was 100% mortality in patients with a hematopoietic stem cell transplant who required mechanical ventilation secondary to acute lung injury with concurrent hepatic and renal insufficiency or hemodynamic instability [23]. Despite this finding, there have been several recent studies that concluded that admission of HM patients to the ICU is appropriate [6, 20, 24].

In an attempt to guide patient care, predictors of poor outcome in HM patients would be beneficial for patients and clinicians when making important medical decisions. In our study, we found that mechanical ventilation and use of vasopressors were predictive of ICU mortality, but not 12-month mortality. Our results are comparable to those of Bird and colleagues who reported that use of mechanical ventilation and multiple organ failures were predictive of ICU mortality [6]. However, they also noted that variables that had been previously associated with mortality, such as neutropenia, transplantation status, and APACHE II score, were not predictive [6]. A recent prospective study similarly found that mechanical ventilation and vasoactive drug use were associated with higher in-hospital mortality but did not comment on ICU mortality [12]. This study also documented the use of hemodialysis in 25.9% of patients and an associated in-hospital mortality rate of 59.2%. We had a lower rate of hemodialysis in our patient population and did not find that hemodialysis was prognostic of ICU mortality.

Other studies have identified factors prognostic of poor patient outcomes to include admission characteristics such as APACHE II score [19, 20], neutropenia [3], type of HM [19], remission status [20], and ICU interventions including mechanical ventilation [25], vasopressor use [19, 20, 25], and use of renal replacement therapy [26]. Interestingly, our study found that having a prior stem cell transplant was not predictive of ICU mortality or any of the measured mortality outcomes with the exception of 12-month mortality. A recent prospective study in hematopoietic stem cell transplant recipients found an overall ICU mortality of 61% [22]. This is higher than our observed ICU mortality rate of 43.5% for this patient subgroup. Another large retrospective review performed in 2009 found that 47% of hematopoietic stem cell transplant patients were discharged from ICU [27]. The differences between our study and previous reports are indicative of the challenges of identifying prognostic variables that can be applied to all patients with HM who are admitted to the ICU. Based on available data, no set of variables has been established to allow for uniform criteria to limit ICU admission in this patient population [4, 24].

An admission to the ICU is a substantial investment in patient care and a limited resource in many hospitals. Patients with a HM have also been shown, on average, to require more costly medical regimens while in the ICU [28]. However, recent guidelines have been published by the Ethics Commission of the French Society of Hematology with regard to the admission of a patient with a HM to the ICU suggesting that an interdisciplinary approach between the hematologist and intensivist should be undertaken when a HM patient presents with organ failure [29]. They propose that transfer to the ICU should be considered or deemed necessary in patients with a HM unless their underlying condition is at a palliative stage or there exists an irreversible condition that is deemed to be end-stage. This proposal is supported by a previous study that demonstrated that patients who survive an ICU admission continue to have no alterations in health-related quality of life when compared to that of the overall cancer population [12]. The findings of our investigation highlight the importance of this concept, as we were unable to determine factors that were uniformly associated with mortality.

This study has the inherent limitations of a retrospective analysis performed at a single center. We included a heterogeneous group of patients in our review, which may limit the validity of our results. We were unable to include well-defined ICU scoring systems such as the APACHE II or SOFA in our analysis, as this information was not routinely recorded through the duration of the study. In our center, there are no established criteria for ICU admission in this patient population, and therefore our study population was determined by physician preference and patient acceptance of advanced life support. It is likely that factors involved in decision-making around the acceptance of ICU admission are important unmeasured variables. In addition, although we are confident that we have captured all the admissions of HM patients in our center, practices that are center specific may impact the ability to generalize our results to other health care settings. Despite these limitations, we believe that this study is an important contribution to the growing body of literature on factors that are predictive of poor outcomes in HM patients admitted to the ICU.

In cases of patients with HM admitted to our ICU, we observed a high rate of mortality (45.6%) which increased to 74.3% at 1 year. Despite analysis of multiple potential variables, only critical illness (vasopressor/mechanical ventilation), postsurgical admission, and multiple myeloma were identified as predictors of patient outcomes. We believe that early discussion between patients and their caregivers about possible outcomes should they require ICU admission is important given the findings of our investigation. Further research is required to determine prognostic variables to aid in the management of this patient population.

## Figures and Tables

**Table 1:** Baseline characteristics of study participants.

Characteristic	*n* = 206 cases
Demographics	
Age (±SD)	51.3 ± 13.6
Male (%)	123 (59.7%)
Type of hematologic malignancy	
Acute leukemia	71 (34.5%)
Chronic leukemia	14 (6.8%)
Lymphoma	69 (33.5%)
Myeloma	21 (10.2%)
Other	31 (15%)
Admission diagnosis	
Hemodynamic	79 (38.3%)
Metabolic	5 (2.4%)
Neurologic	16 (7.8%)
Postarrest care	7 (3.4%)
Postoperative	17 (8.3%)
Respiratory	82 (39.8%)
Other	2 (1.0%)
Median ICU length of stay (IQR)	3.0 (6.0)
Stem cell transplant	92 (44.7%)
Patients requiring readmission	27 (13.1%)
Admission characteristics	
GVHD	33 (13.1%)
Anemia on admission	205 (99.5%)
Neutropenia on admission	103 (50%)
Febrile neutropenia	117 (56.8%)
Thrombocytopenia on admission	178 (86.4%)
Infection on admission	148 (71.8%)
Steroid use prior to ICU	156 (75.7%)
Venoocclusive disease	18 (8.7%)

Data presented as number of patients and % in brackets unless otherwise specified.

ICU: intensive care unit; GVHD: graft versus host disease.

**Table 2:** Interventions during course in ICU.

Intervention	*n* = 206 cases
Vasopressor therapy	117 (56.8%)
Mechanical ventilation	148 (71.8%)
Reintubation	11 (5.3%)
Hemodialysis	36 (17.4%)

Data presented as number of patients and % in brackets.

**Table 3:** ICU patient outcomes.

Outcome	*n* = 206 cases
ICU mortality	94 (45.6%)
30-day mortality	122 (59.2%)
60-day mortality	129 (62.6)
12-month mortality	153 (74.3%)

Data presented as number of patients and % in brackets.

ICU: intensive care unit.

**Table 4:** ICU and long-term mortality by type of hematologic malignancy and previous stem cell transplant.

Hematologic malignancy	ICU mortality	30-day mortality	60-day mortality	12-month mortality
Acute leukemia	35 (49.3%)	45 (63.4%)	49 (69.0%)	57 (80.3%)
Chronic leukemia	9 (64.3%)	10 (71.4%)	11 (78.6%)	12 (85.7%)
Lymphoma	35 (50.7%)	44 (63.8%)	46 (66.7%)	52 (75.4%)
Myeloma	4 (19.1%)	6 (28.6%)	6 (28.6%)	9 (42.9%)
Other	11 (35.5%)	17 (54.8%)	17 (54.8%)	23 (74.2%)
All patients with SCT	40 (43.5%)	50 (54.3%)	54 (58.7%)	61 (66.3%)

Data presented as number of patients and % in brackets.

SCT: stem cell transplant; ICU: intensive care unit.

**Table 5:** Logistic regression predicting ICU mortality and long-term mortality.

Predictor	ICU mortality	30-day mortality	60-day mortality	12-month mortality
Age	1.00 (0.98, 1.027) 0.91	1.02 (0.99, 1.04) 0.18	1.02 (0.99, 1.05) 0.14	1.01 (0.98, 1.04) 0.40
Male	0.83 (0.43, 1.61) 0.58	0.91 (0.48, 1.73) 0.78	1.1 (0.57, 2.11) 0.77	1.52 (0.74, 3.13) 0.26
Malignancy				
Acute leukemia	1.22 (0.46, 3.29) 0.69	1.35 (0.53, 3.46) 0.53	1.87 (0.73, 4.8) 0.20	1.39 (0.47, 4.08) 0.55
Chronic leukemia	3.33 (0.72, 15.46) 0.12	1.8 (0.41, 8.01) 0.44	2.7 (0.55, 13.28) 0.22	1.48 (0.24, 9.11) 0.67
Lymphoma	1.92 (0.71, 5.22) 0.20	1.43 (0.56, 3.65) 0.46	1.56 (0.61, 4.01) 0.35	1.05 (0.36, 3.03) 0.92
Myeloma	0.4 (0.09, 1.82) 0.23	0.25 (0.07, 0.95) 0.041	0.22 (0.06, 0.85) 0.028	0.18 (0.05, 0.72) 0.015
Other (ref)	1	1	1	1
Admission diagnosis				
Hemodynamic (ref)	1	1	1	1
Metabolic	1.34 (0.16, 11.50) 0.79	0.25 (0.03, 2) 0.19	0.4 (0.05, 3.51) 0.41	0.35 (0.04, 3.35) 0.36
Neurologic	1.21 (0.32, 4.63) 0.78	1.58 (0.43, 5.79) 0.49	1.41 (0.38, 5.2) 0.61	0.91 (0.21, 4.01) 0.90
Postarrest care	0.82 (0.14, 4.69) 0.83	0.36 (0.07, 1.94) 0.23	0.31 (0.06, 1.7) 0.18	0.23 (0.03, 1.53) 0.13
Post-op	0.21 (0.05, 0.86) 0.031	0.34 (0.09, 1.23) 0.10	0.3 (0.08, 1.11) 0.07	0.15 (0.04, 0.61) 0.008
Respiratory	0.89 (0.4, 1.98) 0.78	0.77 (0.35, 1.67) 0.51	0.82 (0.37, 1.81) 0.62	0.64 (0.26, 1.59) 0.34
Stem cell transplant	0.77 (0.38, 1.56) 0.46	0.74 (0.37, 1.45) 0.38	0.87 (0.44, 1.73) 0.70	0.46 (0.21, 0.99) 0.047
Necessity for vasopressor therapy	3.73 (1.71, 8.14) <0.001	2.21 (1.05, 4.63) 0.036	2.14 (1.01, 4.55) 0.047	1.52 (0.66, 3.47) 0.32
Necessity for mechanical ventilation	3.42 (1.39, 8.44) 0.008	2 (0.87, 4.58) 0.10	1.67 (0.72, 3.87) 0.23	2.91 (1.14, 7.43) 0.025
Necessity for reintubation	1.24 (0.3, 5.21) 0.77	0.71 (0.18, 2.83) 0.62	0.89 (0.21, 3.84) 0.88	0.72 (0.15, 3.4) 0.68
Necessity for hemodialysis	0.99 (0.409, 2.43) 0.99	2.2 (0.86, 5.64) 0.10	2.52 (0.93, 6.78) 0.69	1.51 (0.53, 4.33) 0.44
Febrile neutropenia	1.1 (0.54, 2.24) 0.78	0.86 (0.44, 1.69) 0.66	0.69 (0.34, 1.37) 0.29	0.81 (0.37, 1.75) 0.59

Data presented as adjusted estimates with 95% confidence intervals in brackets followed by *P* values.

ICU: intensive care unit.
